# United States Veterinary Medical Association

**Published:** 1891-10

**Authors:** 


### UNITED STATES VETERINARY MEDICAL ASSOCIATION.

The Twenty-eighth Annual Meeting of the United States Veterinary Medical Association was held at Willard's Hall, Washington, D. C., on September 15th and 16th, 1891.

### FIRST DAY'S PROCEEDINGS.

The meeting was called to order by the President, Dr. Huidekoper, at half past ten o'clock, A. M. On roll call by the Secretary, the following were present: Drs. Barron, Berns, Bryden, Clement, Dougherty, Faust, Faville, Hinkley, Hitchcock, Hoskins, Huidekoper, Kuhne, Kilborne, Kidd, Lyford, Lowe, Martinet, Michener, Miller, R. A. McLean, Peters, T. B. Rayner, Jas. B. Rayner, Jas. L. Robertson, Swedberg, Thompson, Turner, Walmer, Wende, Weber, Winchester, W. L. Willliams.

Others of the profession were present at the opening. Drs. Jno. W. Gadsden, Wm. B. Werntz, N. Rectenwald, Isaiah Michener, J. C. Walker, H. B. Rayner of Pennsylvania, Dr. J. C. Foelker, delegate from the Pennsylvania State Veterinary Medical Association, Dr. H. A. Meisner, Maryland, and Dr. G. A. Jaman, District of Columbia, Drs. C. B. Robinson, J. D. Robinson, and J. E. Parsons, New Jersey, Drs. A. M. Farrington, Wm. Runge, I. N. Krowl, Dr. J. C. Dustan, delegate from the Veterinary Medical Association of New Jersey, Dr. Wm. Somerville, New York, Dr. W. H. Scruby, Minnesota, Dr. H. S. Hogsett, Virginia, Dr. M. E. Knowles, Indiana.

The President addressed the Association as follows:

*Gentlemen:*

This association has nearly completed the fourth decade of its existence, and within the last decade, we have held meetings, when we looked rather anxiously to know if there would be a working quorum of members present, and regretted that, having come a greater or less distance, that there was not more to be done than the alteration of some of the by-laws, the resurection of some oft-time discussed question on pleuro-pneumonia, and the eating of a dinner. Within the last few years no such problems have been obtrusive; we have gained in the number of our members and in the zeal with which they attend; since the adoption of the new constitution—their last revision—the constitution and by-laws have been let alone and we trust that they will be unaltered for a long time to come; other diseases than lung-plague in cattle have demanded the attention and legislation of the country, and reports and papers have been of sufficient volume and merit to fully occupy our time.

Last year for the second time we varied the itinerary of the association's cycles and left the New England and Middle States, to go West for which we were well rewarded; the older members were shown how large and how active the profession had grown in the vast agricultural districts of the Mississippi, and the veterinarians of that region learned that there existed a United States Veterinary Medical Association, founded, organized and managed for the interest of the profession at large and destined to become in reality as well as in name the representative body of the whole country.

The success of the meeting held last year at Chicago, seemed to demonstrate conclusively that the change from two semi-annual meetings of a day each, to one annual meeting lasting over two or more days had been a fortunate one, but the test and proof of this must lie with the members themselves in the interest which they show in attendance, and attention to the matters which are brought before the Association. The Association has assumed a size and standing now which makes the success of its meetings not only of consequence to the personal comfort and satisfaction of those attending, but the larger meetings and the character of the subjects which are brought before them, have become of public interest and the results of our meetings are reflected over the community, who will judge us and our merits and worth, by the opinion which we ourselves form of each other.

There are doubtless here many members for no fixed or definite reason beyond the good fellowship, and social enjoyment which they know they will find, and we all find here one of the relaxations and pleasures of life, but to insure this for the future imposes a serious duty upon us in regard to the election of members and the retention of those whose actions and methods are detrimental to the profession. We are all apt to be too good-natured in overlooking injurious things which do not seem to affect us individually. It is incumbent upon every member to scan and search the list of applications for membership as critically as he would the records of his own personal associates, and if there exists any proper cause why a man should not become a member, the reasons for it should be placed before the Association. Putting to one side all personal feeling and prejudices, let every man vote for or against the candidates according to their merits as reputable and honest Veterinarians.

I wish to urge upon you the importance of organizing and conducting local, county and state Veterinary Associations, upon principals in harmony with the United States Veterinary Medical Association, and I would like to see it become a requisite condition to qualify a man to become a member of this Association, that he should be a member in good standing of his county and state Association. It should be that when we are asked by anyone, what Veterinarian we recommend in a distant locality that we can answer "any member of the United States Association you will find a reputable practitioner."

But, I am occupying too much of your time, and I will leave it for you to learn for yourselves, how faithfully most of your officers and those to whom you have intrusted the affairs of the Association during the last year have performed their duties.

The Comitia Minora will recommend to you various matters which have been duly weighed and considered by them as for the best interests of the Association.

The Chairmen of their respective committees will report in full the results of their year's labors.

The Secretary, Dr. Hoskins, will make you a report of his work, which I can assure you from personal knowledge has been most arduous and which deserves the most grateful recognition on our part, as to his labors, the greater part of the present enthusiasm of our members is due.

From the reports of the Assistant Secretaries, you will see that they have been unusually active in their work.

Among the papers to be presented, we have that on Barren Mares, by Dr. Lyford; on Rachitis, by Dr. Williams; and others of interest to the practitioner, while in the Report of the committee on Food Inspection, Dr. Bryden's paper on "Foreign Cattle Transportation and its Regulations," and in the discussion of Prof. Liautard's paper on Veterinary Jurisprudence, postponed from last year, we have subjects of national importance, which affect our agricultural products, our health, and our commerce in animals, both at home and abroad.

I beg to thank you, gentlemen, most sincerely for the honor which you conferred on me, by selecting me president of this association for the last year, and as your representative for the moment, for you, I thank the Local Committee of Arrangements, Drs. Walmer, Kilborne, Swedburg, Dougherty, Faville, Clement, and Martinet for their labors which have placed us in such favorable surroundings for a successful meeting which I am sure this will be.

The Secretary read the minutes of the last meeting. Dr. Williams remarked a discrepancy in the minutes, in regard to the membership of the Special Committee on Food Inspection; the name of Dr. Clement had been left off, whilst the name of Dr. Atkinson had remained. The president said that through an inadvertance Dr. Clément's name had been overlooked. Dr. Atkinson was at first named as a member of the committee, and afterwards his name was erased and that of Dr. Clement substituted. The correction was ordered made. The minutes were then received, and, as corrected, approved.

The President called the next order of business, the report of the Comitia Minora, which the Secretary read as follows:

A special meeting of the Comitia Minora held at Willard's Hotel, Monday evening, Sept. 14th, '91, 9. 30 P. M. In the absence of the Chairman, Dr. W. J. Coates, President Huidekoper presided.

The following members were present: Drs. Huidekoper,

Williams, Hoskins, Winchester, R. A. McLean, T. B. Rayner, Lyford and Dougherty.

Absent : Drs. Roberson, Coates and Butler.

The minutes of the last meeting of the Comitia Minora at Chicago were read and approved.

On motion it was moved and adopted that we dispense with the reading of the minutes of the last regular meeting of the Association.

The minutes of the special meeting of the Comitia Minora were read and approved.

The list of applicants for membership then came under discussion ; the first considered were the names for Honorary Membership, and after a thorough examination of the names of Drs. Theobald Smith and Prof. Jas. Law, it was deemed best by unanimous vote, that we do unfavorably recommend.

It was urged that our regular membership being open to gladly receive worthy members of the veterinary and medical profession, that we would heartily consider with favor such names for active membership.

The list for application to regular membership was then considered and the following names were favorably recommended :

Dr. J. T. Ryan, W. H. McKenney, J. T. Donnelly, E. D. Roberts, F. Brenton, J. C. Whitney, W. D. Daniels, E. P. Niles, W. B. Niles, Jno. J. Millar, M. H. Reynolds, John A. Kenny, W. S. Mayo, H. A. Meisner, T. C. McNeil, R. W. Story, M. E. Knowles, J. B. Turner, E. J. Nesbitt, Jos. G. Hill, W. H. Bernmeyer, H. H. Dews, Jas. B. Paige, E. B. Ackerman, W. L. Labaw.

The names of Drs. A. M. Bigelow, W. E. Peterson, Edward P. McKenna, being improperly vouched for, were favorably endorsed, after being vouched for by Dr. J. T. Winchester.

The application of Dr. D. A. Cormack was withdrawn.

The name of Dr. Henry A. White was unfavorably considered, as well as those of Dr. W. T. McCoun, Dr. John Airth.

The name of Dr. G. W. Palmer was not entertained, owing to the filing of an apparent false statement.

The name of Dr. Jno. A. Bell was laid over owing to his application lacking a proper voucher.

For non-payment of initiation fees and dues we recommend that the following names be dropped from the rolls :

Drs. E. M. Berkley, J. E. Gardner, (Albert Hassall), reconsidered. J. S. Culbert, G. Grimshaw, T. C. Herbert, A. Jasma, with instructions to the Secretary that he be directed to remove from his letter-head the claim to membership in this Association. H. C. Klicker, J. Monica, M. O'Connell, G. P. Penninan, Fred. Lamberton, T. E. Maloney, B. G. Orlopp, C. H. Peabody, J. T. Page, W. B. Rowland, S. L. Richards, J. W. Sallade, H. Whitney, Wm. T. Walsh, W. H. Appel, Hara Taka Yokura, E. W. Roche, E. W. Rowland, Geo. H. Roberts, R. L. Tucker, J. P. Wilson, Chas. J. Weidner, Fred. Winant, A. C. Young.

It was recommended that the dues of Dr. J. Penninan be remitted to date.

Information being filed of a violation of the Code of Ethics by Dr. Jno. T. Claris, the Secretary on motion was instructed to request him to make explanation of the same.

It was moved and seconded that we change the wording of the application blank, as follows, strike out "References" and insert "Vouchers for."

On motion it was moved and adopted that we adjourn until 9 A. M. of the 15th.

W. Horace Hoskins, *Secretary.*

On motion the report was received.

*Dr. Peters.* Before accepting that report, do we understand that the Comitia Minora report unfavorably on the honorary membership of Dr. Smith and Dr. Law? I think some explanation is due the Association for such act.

*The President.* That question comes up for action later. You now have before you for your action the first recommendation of the Comitia Minora, which is, that the meeting of 1893, in Chicago, be an International one; that a committee of three be appointed to arrange for it and report upon the same at the next meeting.

*Dr. Clement.* I move that the recommendation of the committee on that subject be accepted. It is very proper that the meeting of 1893 should be held in Chicago, and should be an international meeting. I have not given the subject much thought, but that is my impression just now. Dr. Berns seconded the motion. The question was put, and the motion was agreed to.

*The President.* The next recommendation is in reference to the application blank. Dr. Faust moved that the Association authorize the use of these blanks, which have been adopted by the Comitia Minora. The motion was seconded, the question put and agreed to.

*The President.* The third recommendation of the Comitia Minora is in reference to the action of the Committee on the applications of Drs. Smith and Law for honarary membership.

*Dr. Clement.* It seems to me that we should be rather guarded in this matter. As far as Dr. Smith is concerned (I do not know anything about Dr. Law, never having seen him), he is perfectly willing and anxious to come into this Association as an active member, but so long as his name has been proposed as an honorary member it may be embarrassing, and perhaps not the proper thing to do to turn him down. I think he should feel probably as some of us felt in regard to the medical association; that his presence was not wanted and he would be rather out of place here. So far as he is personally concerned, he has done considerable for the profession, either directly or indirectly, as you chose to put it, in his special line. Whether we agree with his conclusions or not in all cases, the fact remains that he has done considerable work. I think we had better consider this thing fully before turning down a name like that. It would make it rather embarrassing in the future in proposing members. Perhaps some of us would have the names of men we should like to propose, and certainly we should not like to have them refused membership.

*Dr. Winchester.* That calls for an explanation. I speak for myself, and others may do as they like. Dr. Smith is a young man, and there is nothing in our Constitution and By-Laws against his becoming an active member of this association. If he desires to become one, he can go through the regular course, as the rest of us have done, and undoubtedly his application would be favorably received. But to make an honorary member of any one who sees fit to ask that honor I think would be throwing the honor away as a society.

*Dr. R. A. McLean.* There is this further to be said in the line of Dr. Winchester's remarks: I assure you that the Comitia Minora gave thorough examination to Dr. Smith's case, and I have no doubt his name will be considered favorably if it comes through the regular form. We all had to work for our present standing. If an honorary membership in this association were a gratuitous affair it would be an empty honor. We have had men who have worked for thirty years and who have grown gray-headed in the service, who could well be retired and promoted to honorary membership. Let us consider this an honorary degree as the H. F. R. C. V. S. is in the Old Country. Dr. Peters agreed with Dr. Winchester and Dr. McLean on the subject.

*The Secretary.* The question of honorary membership should be one which individual members should consider very thoroughly before they submit the name. We should submit a name certainly that every one is familiar with, and it should be a person from whom every member of our Association had received marked benefit, and he should be a man so situated in life as in some way to be unfit for active membership. We should not consult our personal wishes and desires in such matter to confer upon some personal friend or warm admirer an honorary membership in this Association. That has seemed to be the tendency in several directions which have already come to the notice of the Secretary.

*Dr. Clement.* I quite agree with the spirit of the remarks made by Dr. Winchester and Dr. Peters and Dr. McLean and Secretary Hoskins, and I shall lay emphasis on what the Secretary has just said. But the point I wish to make in particular is that members should be very careful in proposing people for honorary membership in this association unless they have some particular reasons, and no member should propose the name of one, unless he knows all about it. I feel pretty certain that Dr. Smith did not know his name was to be proposed for honorary membership. Honorary membership in this Association should be regarded as of high order to be conferred upon those who have done, or are capable of doing great service for the association and the profession in general. I would suggest that Dr. Smith be informed that the Association would be very glad (although I do not know whether that would be in accordance with the Constitution) to have him as an active member.

*Dr. McLean.* I move that the report of the Comitia Minora in regard to the application of Dr. Smith be adopted. The motion was seconded, the question was put, and the motion was agreed to.

*The President.* The next name to be acted upon is that of Dr. James Law.

*The Secretary.* The Committee unfavorably recommended him on the ground that Prof. Law is not too young a member to be identified with this association, as he is in active participation in the work of the New York State Association, but because of the fact that he had been a member of this Association at a critical time in its history and saw fit to withdraw and shirk his part of the responsibility in sustaining the Association before the country. On that ground it was recommended that his name be unfavorably recommended.

*Dr. Clement.* I move that the report of the Committee be adopted. The motion was seconded, the question put, and the motion was agreed to.

*The President.* The next recommendation is in reference to the list of applicants favorably reported by the Comitia Minora.

*Dr. McLean.* I think that instead of going all over these names that it would be well for the Secretary to cast the ballot of the Association. The President. It is proper that the names be read.

*Dr. McLean.* I move that the Secretary cast the ballot of the Association for the applicants for membership recommended favorably by the Comitia Minora.

The motion was seconded, the question put, and the motion agreed to, and the Secretary announced that he had cast the ballot of the Association in favor of the following :

J. F. Ryan, V. S. *Montreal*, Chicago, Ill. W. L. Williams, voucher.
W. H. McKinney, D. V. S., *Chicago*, Genesee, Ill. M. R. Trumbower, vchr.
J. T. Donnelly, V. S,, *N. Y. Coll.*, Astoria, L., I., N. Y. R. A. McLean, v.
E. D. Roberts, D. V. S., *Chicago*, Jamesville, Wis. Jos, Hughes, voucher.
F. Brenton, V. S., *Ontario*, Detroit, Mich. J. Hawkins, voucher.
J. C. Whitney, V. S., *Ontario*, Hillsdale, Mich. J. Hawkins, voucher.
W. D. Daniels, V. S., *Ontario*, Cardington, O. Wm. R. Howe, voucher.
E. P. Niles, D. V. M,, *Iowa Agr. Coll. Vet. Dept.*, Ames, Iowa, S. Stewart.
W, B. Niles, D. V. M., *Iowa Agr. Coll. Vet. Dpt.*, Ames, Iowa. S. Stewart.
Jno. J. Millar, V. S., *Ontario*, Sioux City, Iowa. J. T. Kennedy.
M. H. Reynolds, M. D., D. V. M., *Vet. Dept. Iowa Agr. Coll.*, *Iowa College of Physicians and Surgeons*, Keosaugua, Iowa, S. Stewart, voucher.
Jno. A. Kenny, D. V. S., *Am. Vet. Coll*, N. Y. City. T. Birdsall, voucher.
W. S. Mayo, M. S., D. V. S., *Chicago*, Manhattan, Kan. Daniel Lemay, vr.
H. A. Meisner, V. M. D., *Vet. Dept. U. of Pa.*, Baltimore, Md. R. S. Huidekoper, Wm. Dougherty, vouchers.
Jas. C. McNeil, M. D,, *Vet. Dept.*, *U of Pa.*, Pittsburg, Pa., J. A. Waugh, voucher.
R. W. Story, V. S., *Ontario*, Princeton, Ill. Dr. Hollingsworth, W. L. Williams, vouchers.
M. E. Knowles, D. V. S., *Am. Vet. Coll.*, Terre Haute, Ind. W. Horace Hoskins, voucher.
J. P. Turner, V. M, D., *Vet. Dept. U. of Pa.*, Fort Niobrara, Neb. W. L. Zuill, voucher.

Alfred M. Bigelow, *Harvard, V. D.* Norwood, Mass. J. B. Winchester, voucher.

Edward J. Nesbitt, *Am. Vet. Coll.*, Poughkeepsie, N. Y. A. Liautard, W. J. Coates, vouchers.

W. E. Peterson, *Harvard V. D.*, Methuen; Mass., F. J. Winchester, voucher.

Joseph G. Hill, *Ontario*, Sennett, N. Y. Nelson P. Hinkley, W. G. Hollingsworth, vouchers.

Edward P. McKenna, *Harvard V. D.*, Woburn, Mass. J. F. Winchester, voucher.

Wm. H. Berkmeyer, *Am. Vet. Coll.*, New York City. E. A. Parsons, T. Birdsall, vouchers.

Henry H. Dews, *Am. Vet. Coll.*, New Bedford, Mass., W. H. Brownell, voucher.

Jas. B, Paige, *Montreal*, Amherst, Mass. F. H. Osgood, voucher.

V. L. La Baw, *Am. Vet. Coll*, Springfield, Mass. A. Liautard, W. J. Coates, vouchers.

E. B. Ackerman, *Am. Vet. Coll.*, New York City. A. Liautard, W. J. Coates, vouchers.

*Dr. Faville.* I move that the Secretary be instructed to cast the ballot of this Association for Drs. A. M. Bigelow, W. E. Peterson and Edward P. McKenna, who being improperly vouched for were favorably endorsed, after being vouched for by Dr. Winchester.

The motion was seconded, the question put, and the motion was agreed to.

The Secretary cast the ballot as instructed.

*The President.* The next clause of the report is the withdrawal of the application of Dr. D. A. Cormack, followed by the recommendation of the committee of unfavorably considered applications of Dr. H. H. A. White, Dr. W. T. McCoun, and Dr. John Airth. On motion the report of the Committee was adopted.

The Secretary read from the report of the Comitia Minora as follows: "The name of Dr. G. W. Palmer was not entertained, owing to the filing of an apparent false statement." On motion of Dr. McLean the action of the Committee was sustained. The name of Dr. John A, Bell was laid over by the Comitia Minora owing to his application lacking a proper voucher. If anybody here would like to vouch for Dr. Bell I would like to have him ready for next year.

For non-payment of initiation fees and dues we have dropped a number of names, They have been given far beyond the time allowed by your By-Laws and Constitution to pay their dues to the Association. I sent to some of these parties nine or ten bills with no apparent effect whatever, and the Comitia Minora recommend that their names be dropped from the rolls. They are Drs. E. M. Beckley, J. S. Culbert, J. E. Gardner, G. Grimshaw, F. C. Herbert and A. Jasme, whom the Comitia Minora instructed the Secretary to direct to remove from his letter-head the claim to membership in this Association; also H. C. Klicker, Fred. Lamberton, J. Morice, T. E. Maloney, M. O. Connell, B. G. Orlopp, G. T. Penniman, C. H. Peabody, G. G. Pearson, I. F. Page, E. W. Roche, W. B. Rowland, E. W. Rowland, S. L. Richards, George H. Roberts, J. W. Sallade, R. L. Tucker, H. Whitney, J. P. Wilson, Wm. F. Walsh, Charles G. Weidner, W. W. Appel, Fred. Winant, Hara Taka, Yokura, and A. C. Young.

*Dr. McLean.* I move that the recommendation of the Comitia Minora be adopted. The motion was seconded, the question put, and the motion was agreed to.

*The Secretary.* It was recommended that the dues of Dr. J. Penniman be remitted to date. He was one of the organizers of the association, and advanced in years, and is so situated that he cannot attend our meetings. Dr. Faville moved that the recommendation be adopted. The motion was seconded, the question put, and the motion agreed to.

*The Secretary.* The Comitia Minora recommended that the salary of the Secretary be increased to $200. I recommended before the Committee this raise in salary. Being one familiar with the arduous character of this position I thought no one was in a better position to urge the matter. I had determined not to allow my name to come before you for re-election. In the last year I have sent out between four and five thousand communications of different kinds and I sent out nine hundred and fifty notices of this meeting in the last six weeks. The work of this Association has kept me out of bed until one and two in the morning. I retired at one o'clock last night and go up at five o'clock this morning in order to put the work in shape for this meeting. It is necessary for me to attend all meetings, and last year I used up my salary and fifty dollars besides in attending meetings. I think as a stimulus to your incoming Secretary that you should raise the salary to $200. The dues and income of the Association reach anywhere from $750 or $800 a year, and probably the expenses of such a Secretary as I have been will not reach more than $400 or $500, and I think it is your duty if you expect the work or this Association to be carried out not as it has been done, but as it should be done, your new Secretary will well earn his $200. *Dr. Faville.* I move to increase the recommendation of the Committee to $250, and if I am in order I will also move that our present Secretary be given a life secretaryship in the Association as well as $250 permanent salary. (Laughter and applause.) *The President.* This increase has been based upon a careful calculation of income and expenses so that it might be perfectly warrantable within the limits of the resources of the Association. *Dr. Faville.* It seems to me that the increased membership of this Association, and our increased income, with the increased labor which the Secretary of necessity must have placed npon him, and all that sort of thing, that $250 is little enough for us to pay. According to our Secretary if his successor is as economical as he has been we will have two or three hundred dollars over our expenses. With the increased salary that the Comitia Minora has recommended there will be left a balance of $200 or $300 in our hands, and I do not think a portion of that money can be better spent than for compensating our Secretary for his arduous labors. I move that the recommendation of $200 be increased to $250.

*Dr. Williams.* I propose that we give a stated salary with his necessary expenses. We hold our meetings now at widening diverted points. Mr. Hoskins had to go to Chicago last year at considerable expense. We do not know who is to be the next Secretary or where the next meeting is to be held, and it occurs to me the better way to arrange this is to allow the Secretary his necessary traveling expenses and then allow him a salary besides —something which he could regard as a salary. According to Secretary's statement his salary has proved a deficit. He has more than expended his salary in attending the meetings.

*The Secretary.* I visited Washington without any instructions from you to look over the work of the local committee here to see that our room was fit for the meeting and that the hotel reception would be such as we should hove. I looked after the points upon which we have heretofore been tripped up. I also visited the New York Association thinking we might draw some of their members and interest them in the National Association. Those things were not necessary for a Secretary to do, but my interest in the matter was such as to do it and I did it at some expense.

*Dr. McLean.* I move that the recommendation of the Comitia Minora be adopted. The Committee went into this quite thoroughly, and until we find out exactly how well our new members are going to stick to us and pay their dues, we feel that $200 is about as much as we could give, although it does not compensate the Secretary form his labor. I would like to add to the report that the Secretary be allowed to draw from the treasury the sum of $50 for expenses incurred by him during the past year, that would leave the salary at $200 and recoup the Secretary for his extra outlay last year.

*The Secretary.* I had recommended this increased salary solely that you might give your future Secretary the necessary stimulus in the matter. Any additional expense paid out by me has been gladly paid. The success of the National Association has been a thing very dear to me for a great many years, and ever since I have been a member. I ask that the amendment in regard to the $50 be withdrawn.

*Dr. Miller.* Nobody knows better than I of the arduous labors performed by our Secretary during the last year. He has spent many sleepless nights in doing the work of this Association. I know that and I also know that he paid out during the last year more than double the amount he received from this association. If he received to-day double the amount of his salary he would not be compensated for the money he laid out for this association. He says in the modesty of his nature that he did all this for the good of the association, and we take his work for it. but we do not think it proper or right that any man should be called upon to do that continuously while he continues as our Secretary, and I think the motion of Dr. McLean will prevail, and I hope the modesty of the Secretary will excuse him from opposing the move. *The President.* The motion of Dr. Faville, that the Secretary's salary be increased to $250 is before the House. *Dr. Faville.* I withdraw the motion. *The President.* You have before you gentlemen an amendment to the recommendation of the Comitia Minora increasing the salary of $200 to $250. There is an amendment pending thereto authorizing the Secretary to draw upon the Treasury for $50 additional towards defraying the expenses incurred by him during the past year. Do I understand this to apply to future Secretaries? (Cries of "No

No"). *Dr. Faville.* The idea is to adopt the recommendation of the Comitia Minora and to add thereto the authority to the Secretary to draw $50 from the treasury to cover expenses already incurred. This does not apply to anything in the future, but to cover outlays in the past. *The President.* I think that comes under new business. You have now before you the motion as to the recommendation of the Comitia Minora in regard to the future salary of the Secretary. The question was put, and the recommendation of the Committee was adopted.

*Dr. Hitchcock.* I rise to a question of privilege touching the question that came up in the report of those members who had just been suspended for non-payment of dues. I find the name of E. M. Beckett. Also when our Secretary read the names of those suspended I think the first name was E. C. Beckett. I would like to know if he was suspended. *The Secretary.* Dr. E. C. Beckett was not considered. *Dr. Hitchcock.* I think Dr. Beckett will be here during this meeting, and if he is suspended for non-payment of dues it places him in an embarrassing position. I think if he comes here it will be with the intention of paying whatever he owes. *The Secretary.* E. C. Beckett was dropped at Chicago. He had in the neighborhood of eleven bills sent him to which he paid no attention, and was dropped at Chicago. *Dr. Faust.* We should consider when our Secretary has given repeated notices and they are not acknowledged that we are treated with contempt, and it is then high time to drop such members.

*The President.* The next order of business is the report of the Committee on Intelligence and Education.

Dr. Peters made the following report:

*Report of the Chairman of Committee on Intelligence and Education of the U. S. V. M. A. for the year ending September, 1891.*

Mr. President and Gentlemen:

As Chairman of your Committee on Intelligence and Education, I have the honor, for the second time, of submitting our Annual Report for your consideration.

Soon after receiving official notice that I had again been distinguished with the confidence of our President by being appointed to the chairmanship of this committee, for another year, I placed myself in communication with the other members of the committee, and after a due amount of correspondence with them, I have compiled the following statement of the results of our labors.

The conditions which obtained at the time of my last annual report, obtain to-day, and what I wrote regarding the status of our profession at that time, applies with equal force at the present. There is nothing in it which I wish had remained unsaid, although there is much which might have been said then that was omitted from lack of time, and for fear of making the material too ponderous to be interesting. It is my intention now to say a few words more upon the subject of education, and to conclude with a few words upon topics to which I think it would be well to call your attention.

So much has been said and written upon the matter of veterinary education in America, that I have no expectation of advancing any new or original ideas, but simply to reiterate and emphasize a few of those that have already been advanced, with the hope that this association will bring all its influence to bear in demanding progressive action and improvement in all the institutions of veterinary learning upon this continent, to which we have to look for future members of our organization.

If we look at the history of medicine in this country, and compare the history of veterinary medicine with it, I do not think that the outlook is so discouraging as many of our pessimistic writers would have us think it is.

It is not such a great many years since most, if not all, of the medical schools on this continent required but a two years' attendance, and when the matriculation requirements were much less stringent in them than at present. As the medical profession grew in influence and wealth the leading schools extended their course to three years, and made the requirements for admission higher, until to-day we hear that next year the Harvard Medical School, and the Medical Department of the University of Pennsylvania are to increase the regular course to four years, as their faculties have decided that three years is too short a period of study in which to acquire a sufficient knowledge of the science of medicine and surgery with which the young practitioner is to be turned loose upon an unsuspecting public. Heretofore American dental degrees have been the only ones in the field of medicine entitled to any consideration in Europe, and dentistry is the only branch of surgery which Europeans have come to this country to study, but this advance in two of our leading medical schools is a step towards securing more recognition for graduates of American medical colleges abroad, or for the graduates of these two schools at any rate, and it is not impossible, nay, it is even probable, that before many more years elapse there are certain branches of surgery and medicine that foreign physicians will find it to their advantage to come to this country to study. This seems to be particularly true when we consider the magnificent hospital and clinical advantages that are now offered in connection with the courses at the leading medical schools in many of our great cities.

It does not seem to me that it is very difficult for us to draw a parallel between the progress of medicine in this country and that of veterinary science; our profession being, perhaps, thirty or forty years behind her older sister. It is but a very few years ago that there were but two veterinary colleges in the United States, both in the city of New York, requiring attendance at but two short winter sessions, and having a matriculation requirement that practically had no existence at all. With the addition of two Canadian schools there were but four for this great continent, only one of which required a three years' attendance, and was regularly a branch of a university ; the other Canadian school being in all respects no better than those in New York. Since 1883 several other institutions of veterinary learning have been established, some like the old two years' schools, requiring no longer a course of study, and hailing all comers with open arms, no matter what their previous conditions, associations or educations may have been. Others connected with universities of different kinds established a three years' graded course, and made a pretence of having some sort of a matriculation examination. How well they have lived up to it those having inside sources of information can tell you much better than I. Then some of our agricultural colleges have established veterinary departments, and even go so far as to grant veterinary degrees.

This is the condition of affairs to-day. It certainly shows many changes within a few years. They do not appear to be for the worse. Let us hope then, that they are for the better.

The recognition of the veterinary profession by the University of Pennsylvania and Harvard University, certainly shows an interest on the part of these institutions of learning in this branch of medicine, even if the conception of the moving spirits in one of them at least, as to what veterinary science is, may be rather crude and visionary.

The course at these two schools, although having the advantage of being graded, greater length, and having a certain standard of admission, is counterbalanced in many ways by the cram course schools, provided the student entering the latter has sufficient perception and education to understand properly what is taught him, he is likely at the end of two years to know quite as much as the student at the other schools does at the end of three years, for the reasons that the faculties at many of the short-course schools have more veterinarians among them, and the student thus has the advantage of the training and ideas of a number of different men, instead of a very few, perhaps the few being the original veterinarian connected with the school with some of those who have graduated under his pupilage, thus leaving it but little better than a one-man institution. Furthermore, the clinical advantages at some of the shorter-course schools are very much better than at the longer-course ones, the hospital practice and out-patient departments being more extensive, the students in one often seeing more surgery, and more interesting cases in two short winters than those in the other would see in three long years. In one instance, at least, the great university establishing a veterinary department went so far as to ignore the existence of any veterinary profession at all in the city where it was established, and in order to get clinical material for its students, and make both ends meet (not having waited until it had any endowment fund) it did ridiculously cheap work to the detriment of existing practitioners, and such of its own graduates as settled near it.

Besides the veterinary schools pure and simple, we have to consider the veterinary departments of Agricultural Colleges, and perhaps I can best do so by quoting from a letter of a member of this committee, Dr. Gerald E. Griffin :

"If the courses are too short, and the examinations easy, in the regular veterinary schools, surely to Heaven it must be a farce indeed to call a man a veterinarian who graduated from an agricultural school, where he is confined to the conservative ideas of one surgeon, who may not intellectually be far above the average, again I protest against the recognition of these schools by the association as veterinary institutes, and against the recognition of their graduates as veterinarians."

While I agree with Dr. Griffin to a certain extent, and think it would be much better if agricultural colleges did not grant veterinary degrees, at the same time they do grant them, and I do not see how we can very well help recognizing them. Their bearers are certainly educated men, they have a much better general education than most of our veterinary graduates, and in many ways it more than compensates for the greater amount of veterinary training that may be given a man who is so ignorant that he can barely write his own name. If the graduate of a veterinary department of an Agricultural College is not fitted for the duties of a practitioner, the great American public is smart enough to find it out, and his pocketbook is the main sufferer, unless, as is occasionally the case, he is an able enough political wire puller to hoist himself into a government office of some kind.

Having tried, in as few words as possible, to describe the condition of our veterinary schools, we have to consider the means for their improvement, and the matter has received quite as much attention from the pens of our veterinary writers as the standing of the schools themselves.

All manner of remedies are suggested, chief among which are endowments for veterinary colleges from the National Government, with governmental supervision, a uniform standard for admission, and a national examining board to examine applicants for membership in the profession. Or, if the central Government could not assume control of veterinary schools without illegally interfering with States' rights, it is proposed that States wherein veterinary schools exist pass laws of co-operation with the National Government to bring about the desired end.

I am one of those who believe that this country is too vast, the varying conditions of separate sections too different, and the requirements of the people of one locality so diverse from those of another, that it would not be policy for the central Government to dictate the management of the schools, besides being an undue interference with the rights of a people who have heretofore been supposed to have the power of thinking for themselves. In the past paternalism on the part of the general Government has not been advocated in this country, and the passage of a few more pension bills will be quite enough of a drain on the national treasury without any assistance from us, although I am well aware that when one lot of pigs are gathered around the trough it sets all the rest to squealing to get there too.

Nearly all of our institutions of learning have been well endowed by the munificence of wealthy people, generally by bequests received after the deaths of the generous individuals, and in this way all the better-known colleges and universities upon this continent have acquired their funds.

It should be our aim in the future to do our utmost to interest those who are fond of fine horses and cattle in the subject of veterinary education with the hope that in time a portion of those interested in the ownership or breeding of valuable live stock may make their donations to some of our veterinary schools, instead of endowing chairs of Greek or Ancient History in classical institutions, or making presents to theological seminaries, for the dissemination of knowledge that they never knew anything about, or took very little interest in during their lives. At present I am afraid that most of them take veterinary surgeons very much as they find them, without stopping to think who they have to thank for their existence, very ready to find fault with them for not knowing more, and never stopping to blame themselves for the veterinarian's lack of knowledge. Worse than that, many rich live stock owners seem to prefer the services of a quack to those of a man who has taken pains to acquire the best education that he can afford.

As to raising the standard of admission and graduation in the veterinary schools as they already exist, I think that is a responsibility that ought to rest chiefly in the hands of our association. It is an impossibility to have a uniform standard in all the schools in the United States, and even if we did, there would be plenty of room for diploma-mills across our northern border, and hordes of ignorant or lazy men ready to attend them, and return as soon as they could possibly obtain a bit of sheepskin, to become the most dangerous and unscrupulous quacks imaginable.

But it should be the feeling of our association that we advise a higher standard of veterinary training, that a man ought to present substantial evidence of a thorough preliminary education upon entering upon a course of veterinary study, and that we favor at least a three years' graded period of teaching.

If we show that these are our sentiments in no uncertain or timid manner, the faculties of those colleges who really have the interest and advancement of our profession at heart will speedily show a disposition to meet our requirements. Then we can decide what schools to recognize, and only allow graduates from them to enter our association. Schools that are run simply to advertise the officials connected therewith, or to act to their pecuniary advantage will continue to exist, and will continue to have students, but we should not recognize them in any way, and advise young men coming to us for counsel to have nothing to do with them.

At present it is of far greater importance that we advocate a higher standard for matriculation, than that we try to bring about any changes in the present courses of study in the various veterinary schools. Students upon entering should have a thorough English education in any event, and we should do all in our power to encourage graduates of classical and agricultural colleges to enter this profession. Given a young man with a good education, keen perceptions, a well trained mind and a desire to learn, and start him upon the right track, and he will acquire the knowledge he desires. If, on the other hand, he is unused to study, is ignorant, so ignorant that he can hardly read and write, much less take notes on lectures or comprehend the information which his instructor is striving to impart, he would never be a thoroughly educated veterinarian, even if he attended a ten years' course instead of two winter sessions of five months each. In this latter class of men are those, to quote from my colleague, Dr. Griffin, "who complain that our veterinary journals are too scientific, and who admire the organ that gives them prescriptions and cure-alls in its columns."

First of all we must insist upon having *educated* men enter our veterinary schools, and it will be time enough when this is accomplished to criticise the curricula and length and number of sessions. It must be borne in mind also that to a certain extent at least "veterinary surgeons are like poets, born and not made."

In order to be really great as a veterinarian, a man must combine two rare gifts. First, he must have that peculiar mental organization which enables him to grasp and understand the art and science of medicine, so well exemplified in the late Dr. Austin Flint, which enabled him to stand at the head of the medical profession in this country during his life. Secondly, he must have that intuitive faculty of understanding animals, and gaining their confidence. A knack that applies to the horse more than to any other animal, and constitutes the difference between a horseman and one who is not. As the late William Herbert, better known as "Frank Forrester," says in his "Hints to Horse Keepers": "To become a perfect judge of a horse requires the observation and attention of half a life time; nor with every man will these be sufficient, for a certain degree of natural tact and talent, or adaptability to the study, is clearly indispensable; and there are some men who, if they were born in a manger, and brought up in a stable, would never become horsemen or judges of a horse."

Either of these great gifts to a degree approaching perfection, is very rare in any man. How much rarer, then, must be the two combined in a single individual?

"Frank Forrester's" words make as good an argument as could be advanced in a few sentences against the old-fashioned idea, that prevails more in England than with us, that the prospective veterinary student should serve a period of pupilage with a practitioner or farmer, in order to learn about animals and their management.

Our Secretary, Dr. Hoskins, in a recent article upon "Uniform Veterinary Education," after discussing various means for alleviating existing evils, suggests that it would strengthen our association, and give it more influence with the veterinary colleges, if legislation could be secured requiring that all candidates for veterinary positions in the army, and upon the Bureau of Animal Industry, and similar offices, be members of our association, it would also in a measure relieve the places from the "bane to-day of our infamous spoils system."

I will weary you no further with the matter of education, although one of my co-workers upon the committee advised that our report would be stronger if I devoted it entirely to this subject, as there are two or three other matters upon which I wish to say a few words.

First and foremost among them is the great medical sensation of the year, Koch's remedy for the treatment of tuberculosis, of which so much was expected, and from which only a great harvest of disappointed hopes was gathered. Even after it was found to be a failure as a remedy it still retained great interest to us as veterinarians on account of its possible value as a diagnostic agent in determining doubtful or suspicious cases of tuberculosis among our bovine patients, but here again it proves to be a disappointment, as in some cases of tuberculosis the animal fails to react to the inoculation, and in other instances where the subject shows a marked reaction to the subcutaneous injection of the material, tuberculosis does not exist. A great drawback in arriving at its true value has been the anxiety on the part of many, if not nearly all, of the experimenters in whose hands it has been placed to obtain the results from its use predicted by Koch, rather than to observe its action in a cool, impartial manner, and accept the facts exactly as they are.

Koch's treatment of tuberculosis consisted in the inoculation of a sufferer from the disease with a ptomaine produced by cultivating the *bacillus of tuberculosis* artificially in a culture media, and is not an entirely original idea, Salmon and Smith of the United States Bureau of Animal Industry have successfully experimented with ptomaines from "hog cholera" cultures as a preventive inoculation for the disease. (See special report on "Hog Cholera" Bureau of Animal Industry, 1889). Professor Rudolph Emmerich has found that in various swine diseases, especially swine erysipelas, if the juices expressed from various tissues or organs of an animal suffering with the disease be rendered sterile and used to inoculate healthy animals, that the animals thus inoculated receive immunity if exposed to the disease. (München Medicinische Wochenschrift, May 12 and 19, 1891).

This method has the great advantage over Pasteur's inoculations with an attenuated virus of the disease in that it is immediately available for use in an outbreak, and that animals not already attacked by the malady may be saved, while in Pasteur's preventive inoculations the animals require inoculating with viruses of varying strength before the disease appears among them.

In this country farmers are apt to be careless and feel no anxiety about the danger of a contagious disease until it actually appears among their animals, when it is too late to apply Pasteur's inoculation, but this new method of inoculating with ptomaines might avail.

This means of preventing disease is based upon the theory that the results of infectious diseases are caused quite as much by the poisons produced by the bacteria, as by the micro-organisms themselves, and that immunity is due to a changed condition of the cells of the body caused by these ptomaines, which gives them the power to resist infection again for a period of considerable duration. Now, if we can introduce the ptomaines without the bacteria producing them we give the animal economy immunity from the action of the disease-producing germs.

Tuberculosis is a peculiar disease. Its tendency is to invade organ after organ, producing irreparable structural changes until death finally comes to the victim's relief, and although many cases appear to recover when placed under proper hygienic conditions, we do not know whether the disease becomes only latent until conditions become favorable for its development, or whether one attack, if the patient really recovers, gives immunity from another. In fact, one attack of tuberculosis seems to predispose to another. Hence, a means of treatment which may be successful in ordinary infectious diseases, where there is a natural tendency towards recovery, and a restoration of diseased organs to a normal condition, may fail of useful results in such a malady as tuberculosis.

While the study of the application of ptomaines as preventives of infectious diseases is yet in its infancy, we must not forget the great boons conferred upon mankind and animals by Pasteur's methods, chief among which is his anti-rabic inoculation. This is brought home to us with especial force because we have now established in New York a Pasteur Institute where the principal work done up to the present time has been the preventive inoculation of persons bitten by rabid dogs, or dogs supposed to have been rabid.

The Pasteur Institute of New York, under charge of Dr. Paul Gibier, with Dr. C. Van Schaick as assistant, and Dr. A. Liautard as consulting veterinarian, was opened for the treatment of persons bitten by rabid animals, February 18, 1890, and during the year ending February 18, 1891, 828 persons presented themselves for treatment. Of these it was demonstrated that in the cases of 643 persons the animals attacking them were not mad, and they were not subjected to treatment.

"In 185 cases the anti-hydrophobic treatment was applied, hydrophobia of the animals which inflicted bites having been evidenced clinically, or by the inoculation in the laboratory, and in many cases by the death of some other persons or animals bitten by the same dogs. No death caused by hydrophobia has been reported among the persons inoculated." (Dr. Gibier's first annual report.) The following is a list by States of those treated :

| | | | |
|---|---|---|---|
| New York, - - - | 81 | Pennsylvania, - - | 5 |
| New Jersey, - - | 27 | Maryland, - - - | 3 |
| Massachusetts, - - | 16 | Missouri, - - - | 3 |
| Connecticut, - - | 11 | New Hampshire, - - | 2 |
| Illinois, - - - - | 9 | Texas, - - - - | 2 |
| Georgia, - - - | 5 | Kentucky, - - - | 2 |
| North Carolina, - - | 5 | Ohio, - - - - | 2 |

One each from Maine, Arizona, Minnesota, Iowa, South Carolina, Nebraska, Rhode Island, Arkansas, Virginia, Louisiana, Indian Territory, and Ontario.

Those who could not afford to pay, were treated free of charge.

This is a good showing for its first year's work, and the amount of misery and suffering saved to humanity by Dr. Gibier and his confrères is incalculable, and yet this institution has no endowment fund of any kind, and no outside assistance whatever, and its charity work comes directly out of the pockets of those connected with it. This is not as it should be, and we, as the veterinary body of this country should assure Dr. Gibier of our sympathy in the great work in which he is engaged, and do all in our power to secure for him the recognition he so richly deserves, and for the Pasteur Institute that pecuniary aid which it stands so much in need of.

One suggestion in our last annual report has already borne fruit in Massachusetts. It was that portion relating to the condition of the United

States Army veterinarian. A friend of our profession, to whom I submitted a copy of my report as chairman of the committee a year ago, who is a member of the present legislature, and very much interested in military matters, presented a bill at last winter's session of the Massachusetts Legislature, providing for the appointment of a veterinary surgeon upon the staff of each batallion of Cavalry or Artillery in the Massachusetts Volunteer Militia, to rank as a 1st Lieutenant, "For," said he, "Massachusetts has taken the lead in a number of military reforms which have afterwards been adopted in the regular army, and this is a chance to bring about a reform in this respect."

The bill became a law, with the result of the appointment of two veterinarians, one to the batallion of Artillery connected with the 1st Brigade, M. V. M., the other to the batallion of Cavalry connected with the 2d Brigade, M. V. M., the first two veterinarians, so far as I know, to hold commissions as officers in the United States. Let us hope they will not be the last. Unfortunately the artillery veterinarian is but a second years' student at the Harvard Veterinary School, but I hope that by another year he will be a full-fledged M. D. V. The Cavalry veterinarian is your humble servant. It is to be desired that members of our own profession and their friends will continue to work to bring about official recognition of our profession in various ways in the different States. What progress has been made towards establishing a veterinary department in the United States Army, you will doubtless be informed by the committee having this matter in charge.

In concluding this report, I believe that the Bureau of Animal Industry should receive a little of our attention. I thought of calling your attention to it a year ago, but my paper then seemed so long that I decided to defer what I had to say until a future occasion, and am now glad that I did so, as it has given me an opportunity to beard the lion in his den, so to speak, which I always prefer to do, if the opportunity permit.

We have connected with the United States Deparment of Agriculture, the Bureau of Animal Industry. Its chief is a veterinarian, and a large number of his assistants are also veterinarians. It is the only department in which the United States Government officially recognizes the veterinary profession in a manner that at all appeals to our self-respect, and as the great veterinary organization of this country, we naturally take much interest in its work and usefulness. We are better able, perhaps, than anyone else to criticise its actions and results, being, as we are, specially educated on the subjects with which it has to deal. We have the same right as the rest of the people to commend the action of our servants, or to find fault with the way in which they conduct their work, besides which, by our special training, we are in a position to feel that we have a peculiar right to show our approval, or our disapproval, as the case may be, of the labors of this Bureau.

Of the practical work of the Bureau of Animal Industry I shall have little to say. It has almost eradicated contagious pleuro-pneumonia from this country, and in time will undoubtedly succeed in its complete extinction. For this service alone it deserves the thanks of the people, and has repaid many times over every cent that has ever been appropriated by

Congress for its support, including all it has expended in other directions. These results could have been obtained by any good veterinarian possessed of tact and administrative ability. When we come, however, to a consideration of its scientific investigations, we cannot say a great deal for its efficiency.

If we review as briefly as possibly the work done in the scientific investigations of swine diseases by the Bureau of Animal Industry, it will be quite sufficient to demonstrate to us the value of its bacteriological work and the credence to place upon any statements emanating from its officials.

If an exhaustive report were written upon the researches in swine diseases in the United States during the past few years, together with all the controversy that they have brought forth, quite a large volume could be easily filled. A year ago, when I thought of referring to this matter in my report, I should have based what I had to say upon an article by J. Amory Jeffries, M.D., which appeared in the JOURNAL OF COMPARATIVE MEDICINE AND VETERINARY ARCHIVES, for December, 1890, entitled "Etiology of Two Outbreaks of Disease Among Hogs," although my report was written before the article appeared in print, I was fully cognizant of its contents, having assisted Dr. Jeffries with the work, and in fact done a portion of it myself. Material which I have since been able to avail myself of only confirms me in the views which I then held, without changing them in any important particular.

The other articles of which I speak, and to which I would refer all interested in the matter, as time will only permit of my presenting the conclusions I have drawn from them, are :

"A Contribution to Our Knowledge of the Cause of Swine-plague, and its Relation to Connected Bacteriological Operations," by Dr. P. Frosch, (Zeitschrift für Hygiene, vol. 9, page 235,) Editors, Dr. R. Koch, and Dr. C. Flugge.

"Upon Our Knowledge of the American Swine-plague," by Dr. Theobald Smith, Chief of the Bacteriological Laboratory of the Bureau of Animal Industry. (Zeitschrift für Hygiene, vol. 10, No. iii., page 480.)

"Reply to the Preceding Work of Dr. Th. Smith, upon 'Our Knowledge of American Swine-plague,'" by Dr. P. Frosch, Assistant in the Institute of Hygiene of the University of Berlin. (Zeitschrift für Hygiene, vol. 10, No. iii., page 509.

Also at a meeting of Scottish Metropolitan Veterinary Medical Society, held in Edinburgh, February 25th, 1891, Mr. Thomas Bowhill, M.R.C.V.S., read a paper upon "Swine Fever." Vide *Veterinary Journal*, May, 1891. To sum up :

Jeffries concludes that Billings' "Swine-plague," and Smith's "Hog Cholera" germs are identical, and differ from those of the disease he has investigated ; and that cultures Smith sent him of his "Swine-plague" germ are identical with the disease germs that he (Jeffries), has been studying, which produce a septic pneumonia in swine that they can communicate to calves, and very probably to lambs, sheep and other animals.

In short, the much-vaunted "Swine-plague" is simply a septic disease which is not peculiar to swine by any means. It is caused by one of a large group of bi-polar organisms, capable of producing similar symptoms in such small experiment animals as are susceptible to them. Jeffries concludes by saying : "But while only two germs of this class are known to infest hogs in the United States, there may be others in Europe, *e. g.* 'Wild seuche.' "

I think that Jeffries' work is particularly accurate and very valuable, and am surprised that it has not attracted a great deal of attention, although it does not seem to have done so.

Dr. Frosch, in his first article, compares the work done by Billings with the work supposed to be Salmon's, and draws the following conclusions :

1. "The bacterium of Salmon's Hog Cholera, and Billings' Swine-plague are identical."

2. "The same is the cause of the American Swine-plague, while the proof of an etiological relation of the bacterium of Salmon's Swine-plague to the first, especially to a second plague of like extent, has not yet been sufficiently demonstrated."

3. "That the bacterium is identical with Selanders's Schweine-pest bacterium" (Selander's Schweine-pest being the swine disease of Sweden and Denmark) "but different from the bacterium of the German Schweine-seuche, chicken cholera, rabbit septicimia and ferret plague."

4. "The ferret disease is caused by a separate kind of bacterium and cannot be grouped with the rest."

Dr. Smith's is a reply to Dr. Frosch's first article.

Dr. Frosch's second paper is a reply to Dr. Smith.

Mr. Bowhill's paper announces that he has found in cases of Swine Fever, in England, a bacterium identical with Billings' Swine-plague germs and that he has sent specimens to Billings, who confirms his discovery.

Here we have two excellent investigators, one in the United States, and one in Germany, confirming the identity of Billing's Swine-plague germ, and Salmon's Hog Cholera germ, and each one acting independently of the other while the third finds the same germ as the cause of the English Swine Fever.

Dr. Billings boldly announces that he found his germ of Swine-plague in July, 1886, among the first pigs that he examined in Nebraska, which had died of the disease.

Salmon, in his report for 1884, discovered a micrococcus as the cause of what he then called Swine-plague. In his report the next year he says it is due to an oval, motile bacterium. Later in some of his replies to his critics he attributes the discovery of this organism to his assistant, Dr. Th. Smith. Dr. Frosch says: "This circumstance not only readily explains the intrinsic contradiction of the reports for 1884 and 1885, but also seems to have influenced Salmons further investigations."

In a special report of the Bureau of Animal Industry upon "Hog Cholera : Its History, Nature and Treatment," issued in 1889, there is a short history of the investigations of swine diseases made in the United States, but we not find any mention of the name of Biliings, although he discovered at once the bacterium which the chief of the Bureau of Animal Industry had been searching for years, and which he probably would not have found for some time, if he had not had the help of an assistant whom he was not generous to credit with the discovery, and so let it pass as his own.

In the report of the Bureau of Animal Industry for 1886, page 20, we find the following statement:

"In view of the results of investigations which have shown the existence of two distinct infectious diseases of swine, perhaps of equal virulence and distribution, a change in the nomenclature becomes necessary in order to avoid any confusion in the future. Since these two diseases have been considered as one in the past, and the name Swine-plague and Hog-cholera have been applied indiscriminately, we prefer to retain both names, with a more restricted meaning, using the name Hog-cholera for the disease described in the last report as Swine-plague, which is produced by a motile bacterium, and applying the name Swine-plague to the other disease, the chief seat of which is in the lungs. This change is the more desirable since recent investigations have shown that the latter disease exists in Germany, where it is called Swine-plague (Schweine-seuche.)"

The following questions propound themselves to us after reading the above:

After speaking of the disease as Swine-plague for several years did the Chief of the Bureau of Animal Industry call Billings' Swine-plague, "Hog Cholera" for the sake of creating confusion? (Thus while apparently ignoring him, at the same time paying him the greatest possible compliment in the power of one man who seems to so admire another.)

If the name "Hog-cholera" was not used in place of Swine-plague for the purpose of creating confusion, why was a septic pneumonia of the pig termed "Swine-plague," unless it was for the purpose of causing still further confusion? When, as we have seen, the disease is not confined to swine, but a little careless study would have shown that pigs could easily communicate it to other species of animals. Dr. Frosch pays the methods of bacteriological study pursued in the laboratory of the Bureau of Animal Industry the deservedly high compliment of doubting any "etiological relation of the bacterium of Salmon's "Swine plague" to the pest, especially to a second plague of like extent."

But Jeffries' work removes all doubt upon this matter, and we know that the Bureau of Animal Industry has found another disease of swine, which is a septic pneumonia, and is not alone confined to swine, and which for some reason or other they choose to term "Swine-plage." Furthermore it is not impossible that one animal may be infected with both maladies simultaneously.

The so-called Swine-plague of the Bureau of Animal Industry is one of those septic diseases due to filth, and is seen chiefly where putrefying city swill is fed, and farmers around Boston find that if the swill is boiled and then fed before there is time for putrefactive process to commence again that they are not troubled with it. In this respect it resembles closely the German Schweine-seuche. If this be a true Swine-plague, make the most of it.

Dr. Smith's article is as I have said in reply to Dr. Frosch's first article. In it he attempts to uphold the work done under the auspices of the Bureau of Animal Industry, and to throw discredit upon the work done in Nebraska, and also to answer the criticisms in Dr. Froschs's first article.

Dr. Frosch's reply to Dr. Smith has its chief interest in his closing sentences. After briefly answering Dr. Smith's remarks, and saying that there is no need of his defending Dr. Billings, as he is abundantly able to defend himself, Frosch ends with: "From the present publication of Smith's, however, which could not be seen in reading the reports of the Bureau of Animal Industry, it is evident that Salmon was not the discoverer of either the 'Hog-cholera' germ or that of the 'Swine-plague' so now we know the condition of things in that regard."

Whether Frosch's feelings of admiration for the honesty and generosity of the pseudo-scientist whose work he supposed he was reviewing when he wrote his first article were equal to his feelings of pity and contempt for the assistant, who was obliged to give the credit for his hard work to his chief, or lose his official head, and yet serve as a pillar for his doughty chief to hide behind in case of an attack, I leave to your imagination.

You will see that Jeffries in his paper gives Smith the credit for the work he has done. It has been no secret to me for the last year and a half as to who was actually conducting these investigations in the Bureau of Animal Industry. Having taken the investigation of swine diseases as a fair sample of this Bureau's scientific labors, are we to be expected to place any dependence upon the accuracy of the statements emanating from its officers concerning such work, especially when they conflict with the results obtained by men like Paquin and Billings, unless the work of the former is confirmed by experiments conducted by independent and unprejudiced observers of recognized ability?

How can we as a profession feel anything but disgraced when we think of the opinions which must be held in Koch's laboratory, the greatest bacteriological laboratory in the world, concerning our Bureau of Animal Industry, and its scientific work?

I do not wish anyone to think that I have taken up the cudgels in Dr. Billing's behalf. Scientific research is the search after truth, and work that is recognized as good abroad cannot be ignored at home, no matter what the personal feelings of one man may happen to be towards another. No one deplores more than I the personalities that so often pervade the writings of the investigator employed by the State of Nebraska, that have done so much to detract from the dignity of his work, which I believe to be really correct and valuable, and it must be borne in mind that blaguardism does not add to the weight of argument. On the other hand a lack of honesty and straightforwardness is equally bad or worse, and modern political methods are not to be tolerated in the conducting of scientific researches.

The former style of writing shows what it is on the face of it. The latter often hides a good deal beneath its surface. One is like the rattlesnake which gives warning when it is about to strike. The other is more dangerous, like the deadly moccasin, which strikes its fangs into its victim without giving any indication of its presence.

If the Bureau of Animal Industry is to be a political organization, why not have its chief simply write the letter of transmissal of his annual report to the Secretary of Agriculture, and have a few true scientists in its employ to work unhampered, and make their own reports upon the questions that they have been studying upon. This at least for the sake of making a more creditable appearance to other civilized nations, if we have no respect for ourselves.

More could easily be added of adverse criticism upon the management of the Bureau of Animal Industry, but enough has been said for the present, and it does not seem advisable to continue this report to too great length.

In conclusion, I wish to heartily express my thanks to my confrères upon this committee for the valuable assistance they have rendered me in obtaining material for this report.

AUSTIN PETERS, M.R.C.V.S.,
*Chairman.*

The Secretary announced that several photographers had made application to take the photograph of members of the convention at no cost to the association, but that such members as wished a copy of the photograph could purchase the same. At this point (forty-five minutes past twelve o'clock) the meeting took a recess until two o'clock P. M.

AFTER RECESS.

At the expiration of the recess the Convention met. The President announced that the next order of business was the report of the Finance Committee. That report went over for the present and the President called for the report of the Committee on Diseases.

*Dr. Michener.* As Dr. Butler, the chairman of the committee, has not sent any communication to me as the second member of the committee, I take it that there is no report to be made.

*The President.* The next report in order is that of the Prize Committee. (No report was offered).

The report of the Special College Committee was called for, but its reception was postponed for the present. The next call was for the report of the Committee on Army Legislation.

*Dr. Miller.* I beg leave to offer an apology. I have been unable to make any headway in the matter of Army Legislation, although I have done considerable work myself unassisted by other members of the committee. I am, therefore, unable to make any report that would be of any particular interest to the association. I shall, however, read that little which I have collected together in my investigation of the matter, and will conclude by making some suggestions for the views of members who may be present as to future legislation.*

*The President.* We are now ready to hear the report of the Publication Committee.

*The Secretary.* Read the report as follows; which was received for discussion:

### PUBLICATION COMMITTEE.

*Mr. President and Fellow Members:*

As Chairman of your Publication Committee I would briefly report that through the work of our stenographer at Chicago, we were enabled for the first time in our history to have a complete report of our transactions. This entire matter has been placed at your command during the year through the kind courtesies of the *American Veterinary Review* and JOURNAL OF COMPARATIVE MEDICINE OF VETERINARY ARCHIVES. The good these journals have done for us cannot be properly estimated at this writing, and their generosity should receive our just recognition. The magnificent work of the *American Veterinary Review* in its special number, containing in entirety the whole work of our meeting of 1890, the most memorable in the association's history, will prove a valuable record on our library shelves for years to come. It seems to me that some such plan as the "Review Special" should be yearly adopted hereafter, as it seems unfair to ask so much space of our veterinary publications, and especially so when appears simultaneously the same matter in corresponding numbers of our journals. The publication committee of the future might well consider the advisability of this suggestion and thus leave to the work of our literary representatives the proper criticism and deductions of our work, that shall lead our efforts to higher and better results.

I want to acknowledge here with profound appreciation the generous gift of the editor of the *Review* in favoring the association with some three hundred and fifty copies of its special edition. A large part of this number has been sent forth to those who were not members of our organization and to other journals and bodies that would be led to an interest in our work as fellow veterinarians and as citizens of various communities where we felt a need of stronger recognition and support.

During the year five hundred copies of list of officers, committees and members were printed, and a copy sent to each member. The remainder were used in calling attention to our existence among members of the profession who should be numbered among our workers. Others were sent to various commercial sources, from which emanate articles of commerce of interest and use to us in our profession, that you all might be kept posted on the entrance into use of new veterinary literary productions, new remedies, new inventions and instruments, all of which I trust would aid and facilitate your daily routine of duties.

Much miscellaneous matter has been put forth by your committee,

* The report will appear later.

consisting of blank applications, blanks for signatures to constitution and by-laws, blanks for notifying members elect, etc., etc., all of which materially aid to a methodical manner for the transaction of our work.

W. HORACE HOSKINS, *Chairman.*

*The President.* The next order of business is the reception of the report of the Special Committee on Central Organized Body.

*Dr. Robertson.* As the second member of that committee I have no thing special to report in regard to the matter. It is a subject hard to understand exactly.

*The President.* The discussion of the reports will come up in their proper order. The next report to be received is that of the Special Committee on Food Inspection.

*Dr. Williams.* What I have to say, if not new to yourselves, is a subject that is new to me. I have to submit the following report :

### NATIONAL AND INTERNATIONAL MEAT INSPECTION.

### REPORT OF COMMITTEE.

Sanitary science in its entirety is one of the broadest, noblest and most ancient of all sciences. From the earliest ages much of the noblest thought, the deepest study, the most sympathetic and earnest endeavors have been designed either directly or indirectly to guard or improve the health of man.

The engineer who effectually drains a malaria-breeding swamp ; the architect who constructs a house with due regard to light and air, and free from disease-breeding drains or refuse receptacles ; the trained agriculturist or horticulturist who detects and destroys unhealthy vegetable food ; the veterinarian who controls or extirpates diseases of animals which by contact or through the use of the meat or milk are fraught with danger to the health or life of man ; the physician who applies scientific measures to control or extirpate disease in man ; and the great masses in close connection with these and many other human labors, all work upon a common ground, for a common purpose—human health and life—striving to vouchsafe to man his biblical three score and ten years with a healthful body and mind.

It is sometimes asserted that this or that profession is the most important of all, but each is vital and it is as difficult to measure or compare their value as it is to fix a price on human health or life.

Our ever changing social, political and geographical environments lead us to view with equally inconstant eyes the role of each of the useful sciences in relation to mankind, The one which proves most attractive under certain conditions commands generally the greatest number of, and most zealous workers, while equally vital subjects are progressing but slowly or lying wholly dormant, until the favored branches have been enthusiastically advanced to a point far beyond that attained by the other correlative sciences, nntil the harmony of the whole is destroyed and the aims of the more advanced sciences are hampered or their progress impeded by the tardiness or deficiency of such branches as may from one cause or another have been suffered to fall far behind. At such times it becomes necessary to find sufficient earnest and competent workers to revive and advance the lagging member and bring it into line with other useful sciences.

During the whole period of human history probably no other vital science has been allowed to drop so far behind its associates, nor suffer so seriously from a long and baneful dormancy as the inspection and control of the flesh and milk of animal intended for human food, until at last the urgent necessity of the situation has forced itself upon the attention of the civilized world, and the demand has gone forth for zealous and efficient workers in the much neglected field, in so effective a manner that already much worthy and highly honorable labor is being done, by a rapidly and ever increasing band of earnest investigators, until we now have abundant promise that meat and milk inspection will soon occupy a highly honorable place in the front rank of the sciences holding a vital relation to human life, health and happiness.

Meat inspection is almost as old as human history and has been fundamentally influenced by religious, social, political and commercial usages. The early Jews enacted meat inspection laws upon an unusually high sanitary plane and enforced them under the sanction of religion and with all the rigidity and zeal of a sacred religious rite. The Jewish meat inspection ordinances apparently rest upon the Mosaic law, and now, centuries after the tables of stone have vanished from the sight of man, these meat inspection laws still seem as indelibly stamped on the mind of the orthodox Jew as they were during the days of Moses, and the civilized world looks with reverence upon the ancient customs and heartily wishes that essentially the same laws could be adopted universally and administered as faithfully and effectually as they were thirty centuries ago.

The necessity for meat inspection is so apparent and well-known to intelligent people that your committee scarcely feel warranted in dwelling even briefly upon this phase of the question. Recent study in connection with the so-called contagious and infectious diseases have demonstrated apparently beyond all chance of doubt that they are each due solely to special living organisms. The contagious diseases of man and animals are in many cases identical and intercommunicable, either by contact or by ingestion of parts of the diseased body by the healthy animal, and it is now a well recognized fact, unqualifiedly endorsed by all who are versed in either human or veterinary medicine, that the ingestion or diseased meat and milk is the direct cause of much disease and death in the human family. The practically universal use of meat and milk as human food in all civilized countries renders the question of their freedom from disease of pressing import to the health of the nation.

Meat inspection possesses great importance also in relation to national economy, as it affords the best and most available means for the prompt detection of the existence of contagious diseases among animals, indicates the location and extent of the infected area and enables the government to institute elaborate study into their nature and causes and the influence of climate, soil, and other environments upon them and to promptly apply remedies for their control or eradication.

In our opinion meat and milk inspection should be carried out primarily in the interests of the intended consumers of the food products and not, as is too often the case, in the interests of the producer.

It should constantly be remembered, however, that the unwarranted condemnation of healthful animal food or that which could economically be rendered sanitary, is a waste of food resources which no nation should tolerate, since by this waste the price of meat is advanced proportionately and thus rendered less and less available as a food to those who stand in greatest need of it—the poorer laboring classes.

The early Jewish meat inspection was carried out with especial reference to Jewish consumers and strictly forbade the use of the flesh of diseased animals as food for their own nation although it would appear from the Mosaic law that such diseased flesh could be sold to other nations, if they desired to buy. Their meat inspection partook of the character of a religious rite, founded, doubtless, on sanitary reasons well known to the Jewish priests. The religious phase of this inspection was probably highly essential to effectiveness, since religious reverence and awe constitute the main source of power in ecclesiastical governments. If an animal was concerned on account of tuberculosis, the greed of the owner could in no way be so effectually controlled and silenced as by the invocation of ecclesiastical law. At the same time under the tribal relations of the Jews he could probably evade the use of a part of the diseased animal for his own food, only with difficulty.

Among the great mass of the human family, where religious sentiment fails to enter into the question of human food, the consumer has slaughtered and inspected the animals intended for use as food in his own family or has relied upon the knowledge and sterling honesty of his butcher.

Recent social, political and commercial changes have seemed to separate farther and farther the consumer of meat from the producer, and a knowledge of its fitness for food becomes more and more difficult to obtain, until under our present environments the meat we obtain at a butcher's stall might appropriately be termed a mystery. An Eastern capitalist, who is in no wise learned in regard to the diseases of cattle, owns a ranch some three thousand miles distant in a western territory, which he visits at intervals of several years and leaves the sale of cattle to irresponsible men who neither know nor care whether they be healthy or not, and from his hands they are shipped two thousand miles to a great slaughter house, where the cattle are bought for the express purpose of slaughtering to sell again, and thence the meat is sold to a wholesale meat dealer in some distant city or perhaps several thousand miles away in some foreign country, then resold again to a retail butcher who finally disposes of it to the consumer. Until within the past few months this process has taken place in this country under the solitary eye of the interested seller, solely from a mercenary standpoint, and leaving the consumer in the most absolute ignorance possible as to the source and sanitary condition of his meat supply. The meat consumers had thus reached the climax of ignorance as to their meat supply, and had attained the pinnacle of danger to which a people can be subjected through the use of diseased meat.

Other countries have in some cases quite old established meat and milk inspection laws and under the goadings of national enthusiasm and love of country, it has been reported that certain of the more highly civilized European nations have ample inspection laws which are effectually administered by a thoroughly trained and organized corps of inspectors.

In the matter of government meat inspection, Germany claims, and perhaps justly, priority in point of effectiveness, but if we are to judge the work of its fruits in the prevention of the transmission of diseases from animals to man; or by the evidence adduced by the highest German authorities themselves, they yet fall far short of an ample law effectively administered. If this can be said of Germany, still more may be asserted of other nations.

The prime obstacle to effective meat and milk inspection has ever been, and will continue to be, the irrepressible and unavoidable conflict between the mercenary interests of the seller and the sanitary interests of the consumer, and upon a scientific and practical adjustment of these interests must meat inspection rest, if it is to succeed.

In essentially all respects the interests of the producer and consumer are identical under a judicious meat inspection, and it is rather due to unscientific and irrational laws and actions that these interests are usually brought into violent conflict. Nutritious meat which is wholesome, or which can be readily rendered healthful, is as much, nay more, of a loss to the poor laborer than to the wealthy stock breeder or meat dealer, for while each carcass excluded from the market serves to increase the profits on those remaining to the seller, they invariably render the procuring of a suitable quantity and quality of meat more difficult for the poor.

Bollinger has well observed, "Were statistics available as to how many persons have died from insufficient nutrition, especially from the want of sufficient nutritive meat we would find a much larger percentage than from the use of the flesh of diseased animals for food." We are thus reminded by this very eminent authority that we should, for humane and national economic reasons, be exceedingly careful to not exclude meats which are or can readily be rendered highly suitable for human food.

We have already stated that meat inspection should be made primarily in the interests of the proposed consumers of such meats. When a Jewish Rabbi, either personally or through a duly authorized party, inspected the meat for a particular tribe or clan, and carefully guarded the interests, from a sanitary standpoint of his own tribe and kinsfolks, his work was accomplished, and the matter concerned only the immediate tribe. Civilization, with its centralization of power, and breaking up of small tribes or clans, and the organization in their stead of vast kingdoms, empires and republics, with the great commercial changes, has rendered the ancient Jewish laws in some of its phases, inapplicable to our present needs. In our country, with our meat producing animals reared at one point, killed at a distant slaughtering center, and then distributed to every part of the union, the nation becomes the consumer of meat and so national meat inspection becomes a necessity. The people of the United States cannot consume the entire meat product of the nation, but have a vast surplus which it is desirable should be sold into foreign countries, where from the density of population or other unfavorable environments, there cannot be sufficient meat produced to supply the necessities of its people. In such event, if we would conform to the proposition that meat inspection should be primarily in the interests of the consumer, it is evident that, if we are to enjoy a large meat export trade, that the inspection of such food products must be so conducted as to properly guard the health and lives of those by whom the meat is to be used. To this end opportunity must be given to the importing countries to know that the inspection is skillfully and conscientously performed.

It is possible that other than sanitary reasons may lead a state or nation to exclude from its markets the food produced of another. We recently witnessed the ineffectual attempts of certain states to exclude dressed meats imported from others by laws apparently inspired by the desire of the affected states, to protect their cattle raisers, and the assertion of Bollinger that, to his mind, more persons die from the want of a due amount of meat than from the eating of diseased meat, would indicate that some of the great meat importing countries of Europe, base their exclusion of foreign meats, upon political rather than sanitary causes.

Your committee, appointed largely for the purpose of continuing the study and discussion of Dr. Schwartzkopff's paper on "National and International Meat Inspection," read before this Association at its last meeting, find it impracticable to review every part of his production at length, We will pass over his remarks as to how meat inspection should be carried out, merely stating that we are in full accord with the author and believe with him that meat inspection should be only undertaken under just and sufficient laws, in conveniently arranged public abbatoirs, and by qualified veterinarians, fully competent to measure the significance of pathological lesions, with which in the course of their duties they will meet, and that the inspection should be carried out by such persons and in a manner to properly guard the health, and command the confidence of the intended consumers.

It has been quite a common custom in many countries requiring meat inspection, to confide that trust to some political hanger-on whose qualifications rest solely uppn political services rendered or promised in the future. It is evident however, to any intelligent person that this duty should be performed by a person well versed in pathology for, as Bollinger has well observed, "meat inspection in its highest sense is applied pathological anatomy."

The recently enacted United States meat inspection laws appear to be, in most respects, adequate and beneficent, and although it is as yet too early to speak of their execution, it is safe to predict that with experience and organization our meat inspection service will prove eminently satisfactory so far as our laws extend.

The law apparently has some imperfections which might be remedied with benefit. The provision in Sec., 7, of this act, which allows "farmers" a peculiar exemption from the general provisions of the inspection regulations and permits him to slaughter diseased animals, and transport the meat into other states, and sell it for human food, apparently nulifies, to a very dangerous extent, the beneficent provisions elsewhere found in the act, and offers inducements to the favored farmer to retain his diseased animals on his farm until slaughtered, and the more apparent evidences of disease are annihilated before offering it for sale.

The failure, perhaps incompetency, of this law to provide for the destruction of diseased meats, and barely excluding them from foreign or inter-state commerce tends strongly to vitiate and deteriorate the *inter-state* or local meat supply. In effect, the United States Meat Inspection law throws back upon the state any animal which may be unfit for human food, and unless the states maintain rigid meat inspection laws (and most of them do not) this provision renders our local meat supply worse instead of better, and benefits only the foreign or extra-state consumer, The Mosaic laws interdicted the use of diseased meats by the Jews themselves but permitted them to sell it to aliens, while our national meat inspection law reverses this order and forces diseased meats upon the local markets.

These provisions will doubtless lead to the enactment of other laws adapted to relieve local meat consumers' from this danger.

The mode of inspection is a question upon which most sanitarians readily agree. There are certain diseases of animals which admittedly render their meat unfit for human food and which being readily recognizable during the life of the animal warrants the inspector in condemning and killing the animal and destroying the carcass. Other animals require a post-mortem examination in order to verify a diagnosis or to discover diseases not discernable during life.

The principal question, the vital one in meat inspection, is what meat shall be excluded from use as human food? The recent meat inspection law of the United States, merely excludes the meat of diseased animals without enumeration or classification, and without dictating what use shall be made of the affected animals or their meat or food products.

Dr. Schwartzkopff, in his previously mentioned paper read before this Association, attempted a classification which to the mind of your chairman, seems quite arbitrary, impracticable and open to considerable criticism.

He divides the whole category of diseased animals into three classes:

(1) Diseases in which animals should be condemned, killed and the carcasses effectually destroyed, viz., Anthrax, Rabies, Septicæmia, Cattle Plague, Glanders, Small Pox in Sheep, Swine-Plague and Hog-Cholera, and Unborn animals.

(2) Diseases in which slaughter may be permitted to ascertain whether the whole or part of the meat is fit for human food, or to be used for industrial purposes, or to be destroyed, viz., Foot and Mouth Disease, Tuberculosis, Actinomycosis Bovis, Icterus, Milk Fever in Cows, Hydrothorax and Ascites, all Diseases which are combined with high Fever, General Emaciation and Debility, for instance, Pneumonia, Enteritis,

Uteritis, etc.; and over-heated and too young animals, which should be kept for further examination.

(3) Diseases only ascertainable after slaughter, and in most cases by the use of the microscope.

Under this head he enumerates the entozoa, known to affect meat-producing animals and one bacteriological disease—actinomycosis suis. How the essayist managed to draw his lines in such a manner is not understood by your chairman.

It is not clear to us why, under his first class—diseases in which animals should be condemned, killing and the carcasses effectually destroyed—he should place glanders, which in many cases cannot be diagnosed except post-mortem, nor hog-cholera, which in the chronic stage may in some cases closely simulate trichinosis, nor can we discover his authority for denominating an unborn animal "diseased" and condemning it to death and destruction.

Again, in his second class he would permit the slaughter of animals affected with tuberculosis, a disease admittedly transmissable to man, and might allow its meat to be sold as food, while under his first class he would exclude swine-plague and hog-cholera, which are certainly not transmissable. Again, he places septicæmia in his first class and destroys the carcass, while uteritis—which is septicæmia beginning in the uterus—he would place in the second class and perhaps allow it to be used as human food. He would kill a cow with milk fever in order to ascertain the suitability of the carcass for human food, when we cannot see what new guide for action would be revealed post-mortem. Under his third class, without stating or suggesting the line of action to be followed as to the use of the meat, he places actinomycosis suis, while all other bacterological diseases, even actinomycosis bovis are placed in the other two classes.

It seems to your chairman that veterinary science is at present sufficiently developed to admit of and to demand a scientific classification of diseased or unhealthy meats, which without enumerating all diseases of animals would serve as a guide in the inspection of meat.

We would not attempt a classification upon the basis of the time at which the disease could be detected and determined upon, but would say that all animals intended for slaughter should first be inspected alive, and if there is sufficient evidence of disease to plainly warrant condemnation, the inspection evidently need go no farther, while in case such evidence is wanting, then permit slaughter and complete the inspection by the necessary post-mortem examination. We would attempt a classification based upon the source or rather the kind of danger which the diseased meat bears for man.

By such a scheme we would have four classes :—

*A.* Parasites of meat, most of which are capable of partial or complete development in the human body.

*B.* Non-transmissable diseases with variations of temperature enumeration or other grave pathological lesions and diseases due to micro-organisms or in the course of which micro-organisms develop, which do not find suitable media for growth in the human body and which, therefore, can only act injuriously upon the human body through their exertions or productions or chemical poison due to their presence.

*C.* Diseases produced by micro-organisms capable of multiplication and growth within the human body and which also produce chemical poisons, which when consumed may produce toxic effects.

*D.* Bacteriological diseases transmissable to man, in which there is no danger from the ingested bacteriological products, but entirely from the living micro-organisms themselves.

We need not enumerate the individual classes under this method of arrangement.

Under Class A. we have a long list of parasitic diseases, which in meats offered for sale are mostly found in a natural state, or matured to the highest point possible in the host e. g., encysted trichinæ, larval forms of tænia, etc. and rarely effect the general condition of the body of the host, and can only injuriously affect the consumer by the ingestion of the living parasite. In such cases, thorough cooking of the meat under official control, renders it safe and only objectionable from a sentimental standpoint.

More rarely it happens that some of these parasites by invading vital organs may cause a general derangement of the system which by producing grave pathological conditions, fever, emaciation, anasarca, etc., as is seen in larval forms of tape worm in the brain ; in strongyli of the lungs and bronchi in cattle and sheep, strongyli of the arteries in the horse, distoma hepaticum of cattle; or by wholesale migration to every part of the body, as in the first stages of trichinosis.

In all such cases the entire carcass, and in those where the parasites, although in a passive state, are yet so abundant as to be abhorent, all infected parts of the carcasses of the affected animals should be not only condemned but rendered innocuous under official supervision since the condemnation of some kinds of parasitic meat and allowing it to be consumed raw by dogs or other lower animals, renders the extermination of such diseases impossible and not infrequently only gives the parasite opportunity to undergo one more stage of development, and return to man in a far more dangerous form than that found in the condemned meat.

Consequently, provision should be made for the complete destruction of these animal parasites before the condemned meat passes from official control.

Under Class B, we place the flesh of animals diseased from a malady not transmissable to man, but which may contain chemical substances, ptomaines or other organic substances, which when ingested may produce serious constitutional disturbances. Included in this class we find a large number of sporadic affections accompanied by variations in temperature, nutrition, etc., of a more or less grave character, as in ordinary pneumonitis, pleuritis, enteritis; etc. It includes also a long list of contagious diseases of animals, among which we find the most destructive epizootics, exerting in most cases great influence on our national economy. Among these are contagious pleuro-pneumonia, hog-cholera, and closely allied diseases, texas fever, and many similar affections. We believe it to be generally conceded that in all this class the flesh of such animal is only rendered dangerous as human food when the disease-processes have attained such a stage as to produce a decided variation of the body temperature, marked emaciation, or other grave pathological conditions, in which cases the meat should be absolutely condemned as human food and only permitted to be used for industrial purposes, after the carcass had been rendered innocuous (in case of contagious disease) to the lower animals.

Class C, might with profit be divided into two rather distinct sub-classes :

1st. A group of what we might term "sporadic" infectious diseases, such as pneumonia, pyo-thorax, pyæmia, septicæmia, uteritis, septic enteritis, pyo-septhæmia or omphalo-phlebitis of new born animals, recent infected wounds, etc.

This includes one of the most important classes of diseased meats in the entire category, although from their nature they are of comparatively little concern to our subject proper—national and international meat and food inspection—because they are ordinarily slaughtered for local use. Bollinger has well observed that of all authenticated cases of death from the consumption of diseased meats, that 80 per cent. were due to the use of flesh from " nothgeschlacteten thieren," or animals slaughtered when death from other causes was iminent, in order to avoid financial loss to the owner. These animals, slaughtered under urgent necessity, belong almost, if not wholly, in this sub-division or rather they constitute it.

In our second sub-class we include the epizootic contagious diseases of animals which are transmissable to man, and which also produces within the animal body ptomaines or other chemical products of a dangerous toxic nature. Rabies, Anthrax, acute and constitutional Glanders, Tuberculosis and Actinomycosis.

The entire carcass of animals coming within this class should evidently be destroyed completely both for sanitary and economic reasons.

Under Class D, we would place three closely allied affections which constitute the most stubbornly contested field in veterinary and medical sanitary science, having engaged the attention of sanitarians for years and promising yet much controversy ere the questions are settled. Upon this class your committee has found it impossible to agree.

This class includes, in our present state of knowledge, three diseases or rather a form of three of the maladies enumerated under the second sub-class of Class C.

They are the chronic local, or perhaps more properly, latent form of glanders, tuberculosis and actinomycosis. They have many characters in common, so much so that we have been enabled only recently to differentiate them. Actinomykosis was long known in one form as glanders of cattle, in other forms as tubercular stomatitis and tubercular enlargement of glands, while in man actinomycosis was known as a variety of tubercular affection. All are peculiarly widely disseminated over the world, and in many cases exceedingly latent or passive, often discernable only by post-mortem inspection, may exist in certain individuals for an almost indefinite time without inducing noticeable constitutional disturbances, they will have a predilection for the lungs and lymphatic glands, gain their admission to the animal economy and effect their extension largely through these. The micro-organisms are highly gregarious in their habits, and have a great tendency to become encysted in various sized cysts with fibrous and fibro-calcareous walls. This encystment renders the enclosed micro-organisms passive, or rather in many cases they retrograde and even perish, and thus in all cases through this encysting process a large portion of the bacilli, sometimes so far as we can discern all of them, become so encysted and render the affection wholly latent, and later these bacilli may largely or wholly perish and recovery of the animal follows, and in two, if not all three, diseases with impunity from future attacks.

All are transmissable to a wide variety of animals and the micro-organisms thrive in vegetable media. In a healthy animal the digestive processes are usually fatal to the etiological moment.

The transmissability of glanders to man and the dangerous character of glanders-affected meat for human food is, we believe, universally conceded.

The contagious character of tuberculosis among the lower animals has been well attested in every possible respect. 1st, By clinical observation. 2nd, By experimental transmission. 3rd, By the production of immunity from subsequent exposure. Its identity in the various lower animals and in man rests upon as thoroughly tested a basis as is recognized in medicine and the inter-transmissability between man and animals has been equally well established. Space forbids that we should consider the question of the fitness of meat of mildly affected tuberculous animals for human food beyond the general conclusions as to the entire class.

The third affection in this class, actinomycosis, constitutes, at present, the most stubbornly contested ground in the whole question of meat inspection from a sanitary standpoint, and it has been found impossible for your committee to agree. Consequently your chairman has felt compelled to present his personal views in the body of the report and has asked Prof. Schwartzkopff to give as completely as possible the contrary phase.

The fundamental point upon which rests the classification herein proposed is the question of the contagiousness of actinomycosis. What constituted a contagious disease? Based upon the derivation of the word and the knowledge of the class of diseases to which it has been applied, it has been held to signify a malady transmissable by contact or approach.

Later developments in medical knowledge have gradually led us to apply this word in a more restricted sense excluding internal and external animal parasites and meaning more particularly those highly contagious affections due to the invasion of vegetable micro-organisms.

We would then say that in the present light of medical science when speaking of contagious diseases in a restricted sense that we apply the term to those affections due to the invasion by a micro-organism, of a more highly organized body, wherein the invading organism finds all the conditions and environments essential to its growth and reproduction and in which the newly generated organisms are capable of attaining the same degree of development and maturity as the initial invading entity and hence becomes capable of transplantation to other analogous organisms. In other words, if glanders bacilli are introduced into the living tissues of a soliped not previously rendered immune, and they multiply and the new organisms attain the same development as that possessed by the bacilli constituting the initial inoculation, then these new bacilli must be equally capable of transplantation with the old and hence under the present meaning of contagion glanders is contagious.

During the discussion of Prof. Schwartskopff's paper on meat inspection he says: "As to the question of actinomycosis, as I said before, theoretically, it is not contagious. . . . . . I do not believe it is contagious, and I base my opinion on my own experience as well as my theoretical studies in handling cattle in the Berlin slaughter houses." The basis for Prof. Schwartskopff's conclusions seem to your chairman somewhat vague.

We fail to comprehend how in ordinary routine work in a Berlin slaughter house, a disease can be classified as contagious or non-contagious. He has not given us any data from his theoretical or practical knowledge of the disease which throws the least light or reason upon his conclusions.

Your chairman, in unison with most veterinarians throughout the world, believes it is contagious and predicates our belief upon the following facts, which will probably be admitted by Prof. S.: (Am. Vet. Rev. Vol. XIV. P. 495)

1st. Bacteriologists, so far as we can learn, universally agree that the micro-organisms found in actinomycosis at lungs, lymphatics, liver, bowels, muscles, etc., of lower animals and man are all identical, with the reproduction of the actinomyces found upon various forms of vegetable food; that in these groups of actinomyces in the affected animal body are to be found micro-organisms identical in morphology, maturity, vitality and reproductive power with the actinomyces found on plants.

2d. It is universally agreed that actinomycosis is due to the invasion of and multiplication in the animal body of these actinomycosis or ray fungi. Under the definition we have proposed for *contagion* these two factors render this disease contagious.

3d. It is generally, if not universally, admitted that actinomycosis originates in animals through the lodgement and multiplication of actinomyces in wounds or abrasions of the skin or mucous membranes—probably in some cases by the inhalation and lodgement of the fungi in the air cells. This fact rests upon abundant and authentic clinical records.

Observations of the disease in man, where the history is traceable, makes it evident in every case that the disease was due to infection through external wounds. Dr. Bodamer, (1) relates a case in a miner where the infection was evidently due to the introduction of actinomyces into a wound. Dr. Schimer, (2) records a case in man referable to wound infection and also Ponfick (3).

Other recorded cases in man are strongly suggestive of inoculation during mastication of actinomyces containing food by means of abrasions of the gums and about the teeth. Compare cases recorded by Dr. Murphy; *N. Y. Medical Journal*, 1885, p. 17; Dr. Conover, *Journal of American Medical Association*, 1885, p. 608; Isarel in *Virchow's Archives*, Vol. lxxiv;

(1) Journal of Comparative Medicine and Surgery, Vol. x., p. 182-199.

(2) Chicago Medical Journal and Exchange, Vol., v. fol., 359.

(3) Aclinomykoses des Menschen, Berlin. 1882.

Ponfick, *De Aktinomykose des Menschen*, Berlin, 1882; Rosenbach, *Centralblatt für Chirurgie*, 1880.

Were the history of inception of actinomycosis of the internal organs of man available, they too would doubtless exhibit distinct evidence of inoculation through a wound, abraded or extremely delicate membrane, after ingestion or inhalation of actinomyces.

Clinical observations of veterinarians in connection with the origin of actinomycosis in animals indicate clearly and beyond contradiction that the disease is due to the translation of the ray fungus from some other organism to a wounded, abraded or extremely delicate surface.

Once the micro-organisms have invaded a wound and have found suitable ground for their growth and multiplication, it is admitted by all scientists that new crops, without known limit as to number, generate, mature and perish, while the disease spreads and augments or sometimes declines in varying degrees,

These propositions are admitted, so far as is known to your chairman, by all scientists whether they believe the disease contagious or non-contagious.

4th. Experimentation, the crucial test as to the transmissability of a disease, fully bears out the above facts. Johne (1), transmitted the disease by inoculation in 75 per cent. of trials with fresh material from a diseased animal.

Ponfick (2) likewise succeeded readily in transmitting the disease in cattle by intra-venous and subcutaneous injections, but failed to transmit it by feeding the fungus to cattle, and had negative results in inoculations in dogs and rabbits. Bodamer (3) records six successful inoculations out of thirteen trials with dogs, cats and rabbits -animals apparently only slightly susceptible.

Israel (4) and Kotter succeeded in inoculating rabbits with actinomyces from man, while Cruikshank (5) had a similar result from inoculating cattle with the fungus from an affected man.

Clinical observations lend strong support to the theory of the transmissibility of the disease from animal to animal. Casewell (6) reports an extensive outbreak of actinomycosis in cattle and hogs bearing strong evidence of indirect transmission from animal to animal by means of food soiled with discharges from actinomycotic abscesses of an affected animal. Your chairman has observed similar instances where 50 to 75 per cent. of a cattle herd was found affected, apparently due in a great measure to transmission in this way from animal to animal. In company with Casewell and other veterinarians (7) we saw an extensive outbreak in the distillery cattle

---

(1) Johne, Actinomykosis Bericht ueber d. Veterinarwesen im Konigreich Sachsen, 1881.

(2) Ponfick ,Die Aktinomykosis der Menschen, eine neue infectiosos-krankheit, Berlin, 1882.

(3) Journal of Comparative Medicine and Surgery, Vol. x. p. 120,

(4) Centralblatt f. Bacteriologie n. Parasitenkunde, B. LII. No. 14, 1888.

(5) Annual Report Agricultural Department, England, 1888.

(6) Am. Report State Board Live Stock Company, Illinois, 1890.

(7) Special Report State Board Live Stock Company, Illinois, (Actinomycosis in Cattle), 1890.

sheds of Peoria, Ill., in 1889, where inter-transmission apparently played a very important role. The extension of the disease seemed due to the fact that badly affected animals were kept in the sheds with the healthy, large abscess about the throat and jaws being irritated and abraded by contact with high mangers, discharged large quantities of actinomyces, containing pus, into the troughs along which flowed the slops to neighboring cattle, and the inoculation of the healthy cattle was favored by their being supplied with very coarse, hard wild hay, which evidently served to abrade the mouth and pharynx.

A long list of the leading scientists of the day might be quoted, who believe in the contagiousness of actinomycosis, such as Bollinger, Ponfick, Johne, Friedberger, Frohner, Rosenbach, Bizzozero, Lindquist, Heller, Peroncito, Ochsner, Crookshank, Fleming, Liautard, Law and others, almost without number. In fact it seems, in so far as your chairman has been able to learn, that contributors alike to standard and current veterinary literature of a recent date all are agreed that the affection is contagious. We understand that Prof. Schwartakopff and other dissenters have given voice to their theories and deductions through the columns of the agricultural or live stock press, and avoided placing their views in form or place where it would come within proper range of scientific criticism. We trust that now, for once, Prof. Schwartakopff and his colleagues will present their non-contagious theory of actinomycosis before this convention, from a scientific standpoint, and permit their arguments to be weighed upon a strictly scientific as well as practical basis. We desire that Prof. Schwartzkopff should fully explain his statement at our last meeting that he predicated his belief of the non-contagiousness of actinomycosis on his theoretical studies, and his practical work in the great abbatoirs of Berlin. What great veterinarians of Berlin taught our fellow-committeeman that actinomycosis was non-contagious while such Germans as Johne, Ponfick, Rosenbach, Friedberger and such veterinarians of the great Berlin Thierartzlichen Hochschule, as Frohner unitedly and without fear or apology denominate the disease as infectious? What facts has he learned in the slaughterhouses of Berlin that demonstrate the non-transmissability of the disease?

Granting the transmissability of actinomycosis and thus supporting the above classification we are prepared to consider the three members of the group jointly in their relation to meat and food inspection. The use of the flesh of solipeds has not gained sufficient popular sanction in this country to warrant us in consuming your time by referring to glanders.

Admitting the identity of human tuberculosis and actinomycosis with these two diseases in lower animals and the proof of their transmissability, the question which confronts us is, what, if any parts of affected animals are safe for human food or can be readily made inoculous?

Upon this question there is a great variation of opinion, rendering any definite conclusions, which would be acceptible at all, extremely difficult, if not impossible.

We have taken the stand that in these chronic or passive forms or disease, the sole danger to the consumer of the meat or milk of affected animals exists in the living, virile micro-organisms so we are at once brought to enquire in what parts of the animal body do the germs exist and in what parts are they absent.

Many contend that only those parts which are evidently affected should be condemned and destroyed. This is apparently the idea of Dr. Schwartzkopff in relation to tuberculosis in his remarks on his paper of last year, but a careful study of his utterance leaves one in doubt as to his real belief.

The warrant for the custom of condemning and destroying parts evidently affected with one of these maladies and allowing the remainder of the carcass to be used for human food must rest upon one or two propositions: 1st, that the micro-organisms exist only in those parts where their existence is plainly evidenced by the agglomerations or, 2nd, upon the theory that meat from a diseased animal must be considered innocuous until the presence of the infecting element in the part in question has been fully demonstrated.

It must be admitted with regard to the first position that these germs may exist in agglomerations in parts of the animal not usually seen by the inspector, *e. g.* meningeal tuberculosis. There is further very good evidence that the germs of these maladies are transmitted from part to part along the natural course in the lymphatics. This is very nicely shown clinically in actinomycosis in man and cattle. In the latter the origin of actinomycosic abscesses in the lymphatic glands in the region of the throat can frequently be clearly traced post mortem to an initial inoculation in the pharynx which has mearly left sufficient trace of invasion to demonstrate the role which this part has played as the point of inception of a disease which is to find a suitable field for development only after the etiological moment has traversed for a comparatively great distance through the channel of a small vessel. We observe this lymphatic transmission most beautifully in actinomycosis of cattle, beginning at or near one of the feet and slowly but steadily moving along the course of the lymphatic vessels, destroying by suppuration the glands, some of the germs even succeeding in passing these sentries ere their destruction prevents further progress, continue their ravages toward the central portions of the body. Evidently the actinomyces are usually some distance in advance of macroscopical pathological changes and we cannot judge to what extent a part is invaded except by an utterly impracticable and laborious microscopical examination with but little warrant of reliability.

The migration of tubercle bacilli and actinomyces through the blood vessels seems to be proven beyond reasonable doubt.

It is true that with but a few years study of these diseases we have recorded but few evident cases of such transmission but we feel that they quite suffice to overcome negative testimony although possibly far more voluminous.

Friedberger and Frohner (1) say that it seems possible that actinomycosis can be disseminated by means of the blood, while in case of tuberculosis (2) they assert that it may and does extend in diverse ways. 1st, By the lymph channels; 2nd, By continuity or contiguity; 3rd, By the blood

(1) Pathol. u. Therape. d. Hausthiere, Bd. II, s. 545.

(2) Ibid., s. 505.

circulation. This blood infection and extension happens by the rupture into blood vessels of tubercular nodules or tubercular affection of the vessel walls. It is well shown when the bacilli are excreted in the milk from an apparently healthy udder. Hamburger (1) cites a case of actinomycosis in a colt one and one-half years old contracted presumably during decubitus through wounds produced by splints applied to correct rachitic deformity of the legs, in which in bones and cartilage and finally in the arteries themselves were found actinomyces.

We are forced to conclude therefore that once an animal is affected in a local or passive form with one of this group that we have no reliable means at our command for determining that apparently healthy parts are in reality sound.

We believe further that it is the duty of the meat inspector rather to be able to say if meat is *fit* for use as food than *unfit*. Where question is raised as to the fitness of given food, we hold that the consumer should be granted the benefit of the doubt, and that the inspector should be able to say unreservedly that the meat is wholesome before he permits it, upon his sanction, to be sold for human food.

The recently enacted United States meat inspection laws are as precise, with all their brevity, as can well be devised. It makes simply and only two classes of meats—those from well and diseased animals. The former is passed as wholesome, the latter rejected.

This is the true, safe line, and affords between the flesh of animals dead from disease (which all civilized people abhor as food) and that of healthy animals a neutral ground, which should act as an effective barrier to the use, though the greed of sellers of this repulsive and dangerous food which with our present laws is yet rendered possible in our local markets.

The meat of animals slaughtered at a time when death from disease is imminent, cannot be considered preferable to those which have died from the ailment. What possible difference can it make from a sanitary standpoint if a cow with parturient apoplexy is slaughtered or allowed to die from the disease one half hour later?

The neutral ground—disease—between health and death is one which the meat inspector should enter with caution, and so we do not hesitate to assert our belief, predicated upon the foregoing reasons, that the meat of diseased animals of this group should not, under ordinary conditions, be passed as wholesome food.

As we suggested at the outset, however we are equally firm in our belief that from the standpoint of humanity and national economy we have no right to destroy wantonly and uselessly such an enormous food supply as the unqualified destruction of these meats would entail. Since the whole danger in the consumption of these meats rests upon the presence, or possible presence within the parts used as food of living, transplantable micro-organism, it is evident that any process to which it can be subjected without destroying the nutritive value, and yet effectually and beyond all doubt destroy the micro-organism, will render the flesh *sound* from a strictly sanitary standpoint. Consequently, we would say that the apparently

(1) Holl Zeitchrift f, Thierheilkunde, etc., Bd. 16, Lf. 2 and 3.

healthy parts of such diseased animals should be thoroughly and effectively cooked (boiled) under official supervision and then passed as sound meat, while the evidently affected parts should be destroyed.

Such a course evidently renders these meats equally safe to that of the healthiest animal, or perhaps even more so, for we cannot at all time discern what insidious disease be lurking unseen in an apparently healthy body.

We have refrained from dwelling on all the phases of the subject of meat inspection as suggested in Dr. Schwartzkopiff's excellent paper of a year ago because time would not permit, but have confined our consideration mainly to those questions which seemed to us most in dispute and which would, in our beiief, lead to the most spirited and profitable discussion. Respectfully submitted.

W. S. Williams, *Chairman.*

The report was received for discussion.

*The President.* We will now have the Secretary's report.

The Secretary read his report as follows :

